# Bilateral Subarachnoid Hemorrhage and Bilateral Intracranial Hemorrhage With Reversible Cardiomyopathy During Dobutamine Stress Echocardiography

**DOI:** 10.7759/cureus.14725

**Published:** 2021-04-27

**Authors:** Khuzema Ghafoor, Hafiz U Ghafoor, Somwail Rasla, Angelis Dimitrios

**Affiliations:** 1 Internal Medicine, Atrium Health Navicent, Macon, USA; 2 Cardiology, Saint Vincent Hospital, Worcester, USA

**Keywords:** dobutamine stress echocardiography, subarachnoid hemorrhage, intracranial hemorrhage, cerebrovascular accident, hypertension, headache, takosubo cardiomyopathy

## Abstract

Dobutamine stress echocardiogram (DSE) is routinely used in the clinical assessment of patients with known or suspected coronary artery disease (CAD). DSE can cause serious complications including cerebrovascular accident (CVA). Even though the incidence of CVA associated with DSE is very low (<0.01%),it can be life-threatening or cause significant morbidity. We present a patient who developed acute multifocal intracranial hemorrhage (ICH) and subarachnoid hemorrhage (SAH) during the DSE.

A 39-year-old female with no prior cardiac history presented to the outpatient echocardiography lab for DSE. She had a blunted heart rate response with increasing dose of dobutamine 30 μg/kg/min and was given one milligram of atropine. The patient complained of frontal headache, nausea, and severe dyspnea. Computed tomography head showed acute multifocal bilateral SAH, and left frontal and right parieto-occipital ICH.

Hypertension is one of the risk factors for ICH and dobutamine infusion can exacerbate severe acute hypertension, which can cause acute intraparenchymal hemorrhage. Even though the risk of ICH associated with DSE is extremely low, there should be increased vigilance if there is development of severe acute hypertension, and the operator should keep a low threshold for further evaluation if the patient develops neurological symptoms.

## Introduction

Dobutamine stress echocardiogram (DSE) is considered more sensitive for diagnosing coronary artery disease (CAD) than other stress modalities [1]. It is routinely used in the clinical assessment of patients with known or suspected CAD. DSE can cause serious complications including cerebrovascular accident (CVA). Even though the incidence of CVA associated with DSE is very low (<0.01%) [2], it can be life-threatening or cause significant morbidity. There is a paucity of cases of CVA during DSE [3, 4]. We present a patient who also developed acute multifocal subarachnoid hemorrhages (SAH), and intracranial hemorrhage (ICH) during DSE. The patient was treated conservatively because she was neurologically intact and the workup showed no evident cause of the bleed.

This article has been previously published to a preprint server: https://yvm2020.authorea.com/doi/full/10.22541/au.159164718.87898312.

## Case presentation

A 39-year-old Hispanic female with no prior cardiac history and not on any antiplatelet or anticoagulant agents at home, presented to the outpatient echocardiography lab for DSE as a workup for her persistent atypical chest pain on exertion. The patient had been well until one month ago when she started feeling substernal pressure sensation, which prompted her visit to a primary care physician. She underwent an exercise stress test that was inconclusive, given the non-specific electrocardiogram (ECG) changes with poor exercise capacity. Her persistent atypical chest pain prompted her primary care provider to refer her for a pharmacological stress test. 

Prior to the day of the test, the patient complained of a nonspecific frontal headache that ultimately resolved with one dose of Tylenol 350 mg. Vitals signs before the stress test were: resting heart rate (HR) 66 beats per minute (bpm), blood pressure (BP) 150/70 mmHg, respiratory rate 22 breaths per minute, and oxygen saturation 100% while she was breathing ambient air. As per protocol, dobutamine infusion was initiated at 10 μg/kg/min, subsequently increased by 10 μg/kg/min at 3-minute intervals. She had a blunted heart rate response with increasing dose of dobutamine, and her vitals at 30 μg/kg/min were HR 78 bpm and BP 161/88 mmHg. One milligram of atropine was given to get the desired response, and her HR increased to 175 bpm and BP to 240/150 mmHg. The infusion was stopped immediately due to the hypertensive response. Images were obtained at peak heart rate and in recovery. 

**Figure 1:**
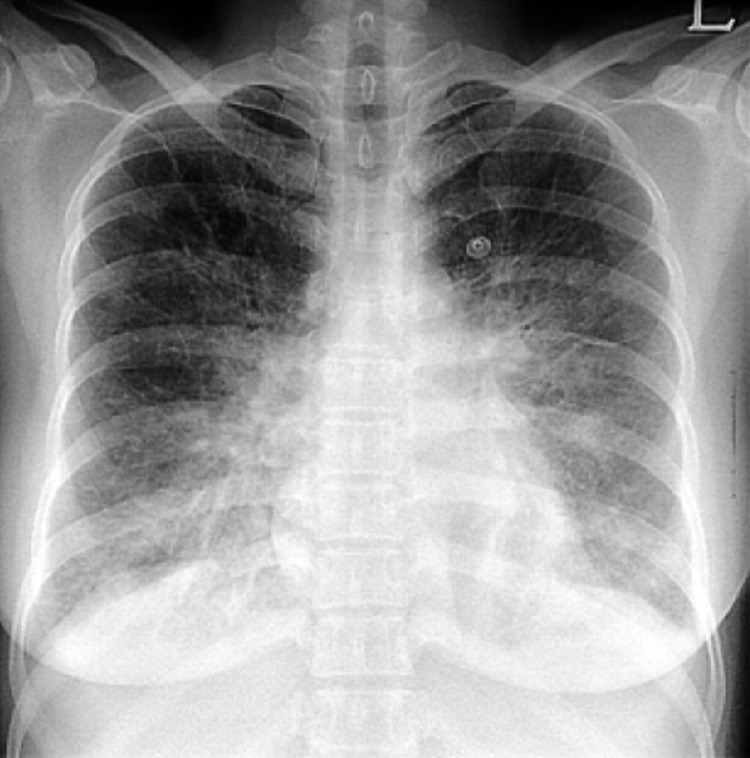
Chest X-ray showed pulmonary edema

The patient complained of frontal headache associated with nausea, worse than what she had in the days before the test. She also started to complain of severe dyspnea with increased oxygen requirement of up to 5 liters per nasal cannula. The patient was given sublingual nitro 0.4 mg and two doses of metoprolol 5 mg intravenous and promptly transferred to the emergency department. CT head showed acute multifocal bilateral SAH, left frontal and right parieto-occipital ICH without midline shift. She was admitted to intensive care for ICH management. Her chest X-ray showed significant pulmonary edema (Figure 1). 

**Figure 2:**
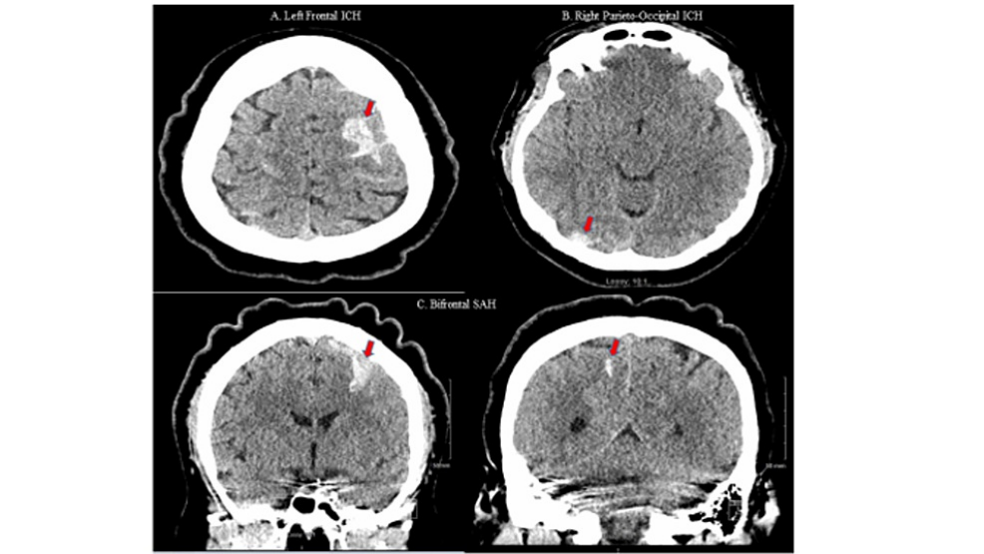
CT Head demonstrating (A) left frontal intraparenchymal, (B) right parieto-occipital intraparenchymal, and (C) multifocal bilateral subarachnoid hemorrhages (arrows)

Non-contrast CT head revealed acute multifocal bilateral SAH and left frontal and right parieto-occipital ICH without midline shift (Figure 2).

CT angiogram of head and neck followed by catheter-guided cerebral angiography showed no aneurysm, arteriovenous malformation (AVM); distal left middle cerebral artery (LMCA) branches showed some narrowing which improved with intra-arterial verapamil. DSE findings were suggestive of severe global hypokinesis (Video 1, Video 2) and transthoracic echocardiogram (TTE) showed resolution of apical hypokinesis (Video 3).

**Video 1:** Apical 4-chamber DSE findings of hypokinesis of distal anterior, the apical and distal septal wall at peak stress with possible augmentation of the base and her post-stress images reveal left ventricular global hypokinesis DSE: dobutamine stress echocardiogram

**Video 2:** Parasternal long-axis DSE findings of hypokinesis of distal anterior, the apical and distal septal wall at peak stress with possible augmentation of the base and her post-stress images reveal left ventricular global hypokinesis DSE: dobutamine stress echocardiogram

**Video 3:** Transthoracic echocardiogram showing resolution of global hypokinesis

The patient was evaluated by Neurology and Neurosurgery and a decision was made to treat conservatively since there were no significant neurological symptoms and there was no aneurysm, arterio-venous malformation (AVM), or active bleed during cerebral angiography. Also, the distal LMCA branches showed some narrowing which improved with intra-arterial verapamil suggestive of reversible cerebral vascular syndrome (RCVS).

The patient remained free from symptoms and a repeat CT head showed resolution of the bleed. She was started on verapamil 80 mg TID by her primary neurologist with no further episodes of headache.

## Discussion

Indications for DSE include diagnosis of CAD, risk stratification of patients with chronic CAD, acute or chronic myocardial infarction (MI), and valvular heart disease. Dobutamine stress echocardiogram has been known to cause serious complications even though it is considered a safe stress modality. These severe complications include myocardial ischemia, myocardial infarction, atrial and ventricular arrhythmias, hypertension, hypotension, myocardial rupture, and CVA [2].

Hypertension is one of the risk factors for ICH [5]. Intracranial hemorrhage associated with hypertension is known to involve small penetrator arteries [5]. Dobutamine infusion can exacerbate severe acute hypertension, which can cause acute intraparenchymal hemorrhage. The first reported case of ICH associated with DSE was a patient on chronic anticoagulation for a prosthetic mitral valve [4]. In the second case, ICH occurred immediately following DSE that resulted in uncal herniation in the absence of anticoagulation [3]. But all cases had an exaggerated hypertensive response to dobutamine. Furthermore, in a large registry on the safety of DSE, only three patients suffered a stroke, but the mechanism of stroke was not reported [6].

In a systematic review, DSE has been shown to induce transient cardiomyopathies including stress cardiomyopathy with apical ballooning in 95% of the study patients [7]. Various mechanisms include catecholamine excess have been postulated for wall motion abnormalities in cardiomyopathies. Cardiomyopathy appears to be associated with several acute neurological diseases like SAH. Stroke is linked to cardiomyopathies by a dual relationship since it may induce cardiomyopathy by catecholamine release and cardiomyopathy itself may be complicated by left ventricular thrombi leading to stroke [8].

In addition to ICH, our patient had features suggestive of cardiomyopathy based on the DSE findings of hypokinesis of distal anterior, the apical and distal septal wall at peak stress with possible augmentation of the base, and her post-stress images reveal left ventricular (LV) global hypokinesis (Video 1, Video 2). However, the subsequent repeat TTE showed resolution of global hypokinesis (Video 3).

Our patient developed ICH due to the exaggerated hypertensive response to dobutamine and was diagnosed with reversible cerebral vasoconstriction stroke (RCVS) after the vaso-reactive testing was done. Calcium channel blockers are shown to be beneficial for the management of the vasospasm.

## Conclusions

The significance of this case, like the others, lies in advising caution for patients undergoing DSE with systolic BP >180 mmHg during the procedure and HR exceeding 85% of the maximal predicted for age. Caution should be taken even if this effect was transient in an otherwise healthy young patient as illustrated in this case. The American College of Cardiology/American Heart Association recommend terminating a treadmill test for a systolic BP >250 mmHg or diastolic BP >115 mmHg during the test.
